# BRD4-targeted therapy induces Myc-independent cytotoxicity in Gnaq/11-mutatant uveal melanoma cells

**DOI:** 10.18632/oncotarget.5179

**Published:** 2015-09-05

**Authors:** Grazia Ambrosini, Ashley D. Sawle, Elgilda Musi, Gary K. Schwartz

**Affiliations:** ^1^ The Herbert Irving Comprehensive Cancer Center, Columbia University Medical Center, New York, NY, USA

**Keywords:** BRD4, JQ1, Gnaq/11, Bcl-xL, Rad51

## Abstract

Uveal melanoma (UM) is an aggressive intraocular malignancy with limited therapeutic options. Both primary and metastatic UM are characterized by oncogenic mutations in the G-protein alpha subunit q and 11. Furthermore, nearly 40% of UM has amplification of the chromosomal arm 8q and monosomy of chromosome 3, with consequent anomalies of *MYC* copy number. Chromatin regulators have become attractive targets for cancer therapy. In particular, the bromodomain and extra-terminal (BET) inhibitor JQ1 has shown selective inhibition of c-Myc expression with antiproliferative activity in hematopoietic and solid tumors. Here we provide evidence that JQ1 had cytotoxic activity in UM cell lines carrying Gnaq/11 mutations, while in cells without the mutations had little effects. Using microarray analysis, we identified a large subset of genes modulated by JQ1 involved in the regulation of cell cycle, apoptosis and DNA repair. Further analysis of selected genes determined that the concomitant silencing of Bcl-xL and Rad51 represented the minimal requirement to mimic the apoptotic effects of JQ1 in the mutant cells, independently of c-Myc. In addition, administration of JQ1 to mouse xenograft models of Gnaq-mutant UM resulted in significant inhibition of tumor growth.

Collectively, our results define BRD4 targeting as a novel therapeutic intervention against UM with Gnaq/Gna11 mutations.

## INTRODUCTION

Aberrant epigenetic regulation plays a central role into the genesis of cancer [1]. BET inhibitors are emerging therapeutics in oncology that specifically disrupt the interaction between BET proteins and chromatin, resulting in the inhibition of cancer growth [2, 3]. The BET family of proteins, including BRD2, BRD3, BRD4, and BRDT are chromatin readers containing two tandem amino-terminal bromodomains that bind to acetylated lysine residues on histone tails. Here, they direct the assembly of nuclear macromolecular complexes that regulate key biologic processes, including DNA replication, chromatin remodeling and transcription [2, 4]. In particular BRD4 was shown to associate to a protein complex that included P-TEFb and to stimulate RNA Polymerase II-dependent transcription [5]. The small molecule JQ1 is the first generation of BET specific inhibitors which competitively displaces BRD4 from acetylated histones, resulting in the suppression of c-Myc [2], and c-Myc-dependent target genes [6–9]. BET inhibition proved to be highly effective against hematopoietic cancers [4, 6, 8, 10], as well as a subset of lung adenocarcinoma cell lines [11], glioblastoma [12] and medulloblastoma [13]. Bromodomain targeting in cutaneous melanoma inhibited the expression of several BRD4-regulated genes, including c-Myc, SKP2 and ERK1 [14]. These studies also demonstrated that although BET inhibitors influence predominantly the *MYC* transcriptome, other genes undergo expressional changes and simultaneously contributed to the decrease of cell viability.

Uveal melanoma (UM) is the most common primary intraocular malignancy of the adult eye. The median survival after diagnosis of metastatic disease is 3.6 months, with a 5-year cumulative survival of less than 1% [15]. UM is biologically distinct from cutaneous melanoma, as 85% of primary and metastatic UM carry oncogenic mutations of G-protein α-subunits q or 11 [16, 17], and have a high tendency to metastasize to the liver [18]. Recent efforts in the understanding of the biology of UM have outlined therapies that target mutant G-protein signaling [19]. Nevertheless, there is a compelling need for effective therapeutic strategies to manage this disease. UM are also characterized by genetic abnormalities, including the amplification of the chromosomal arm 8q and monosomy of chromosome 3, which are significantly associated with poor prognosis [20, 21]. The oncogene *MYC* is located on 8q24.1 and results amplified in nearly 40% of UM [22]. This transcription factor is involved in the transcription of genes regulating cell proliferation, cellular metabolism and survival [23], and its elevated expression correlated with larger tumor size of UM [22, 24].

In this study, we investigate the potential therapeutic effect of the BET inhibitor JQ1 in UM cells. We found that JQ1 induces cell cycle arrest and apoptosis, especially in cells with Gnaq/11 mutations. Using microarray analysis we identified a large set of genes modulated by JQ1 that may account for the differential effects observed in mutant versus wild-type cells. In particular, genes involved in the regulation of apoptosis and DNA repair seem to play role in UM tumor growth. These observations support the evidence that BET inhibition represent a promising therapeutic approach for UM with Gnaq/11 mutations.

## RESULTS

### JQ1 inhibits viability of UM cells

**Figure 1:**
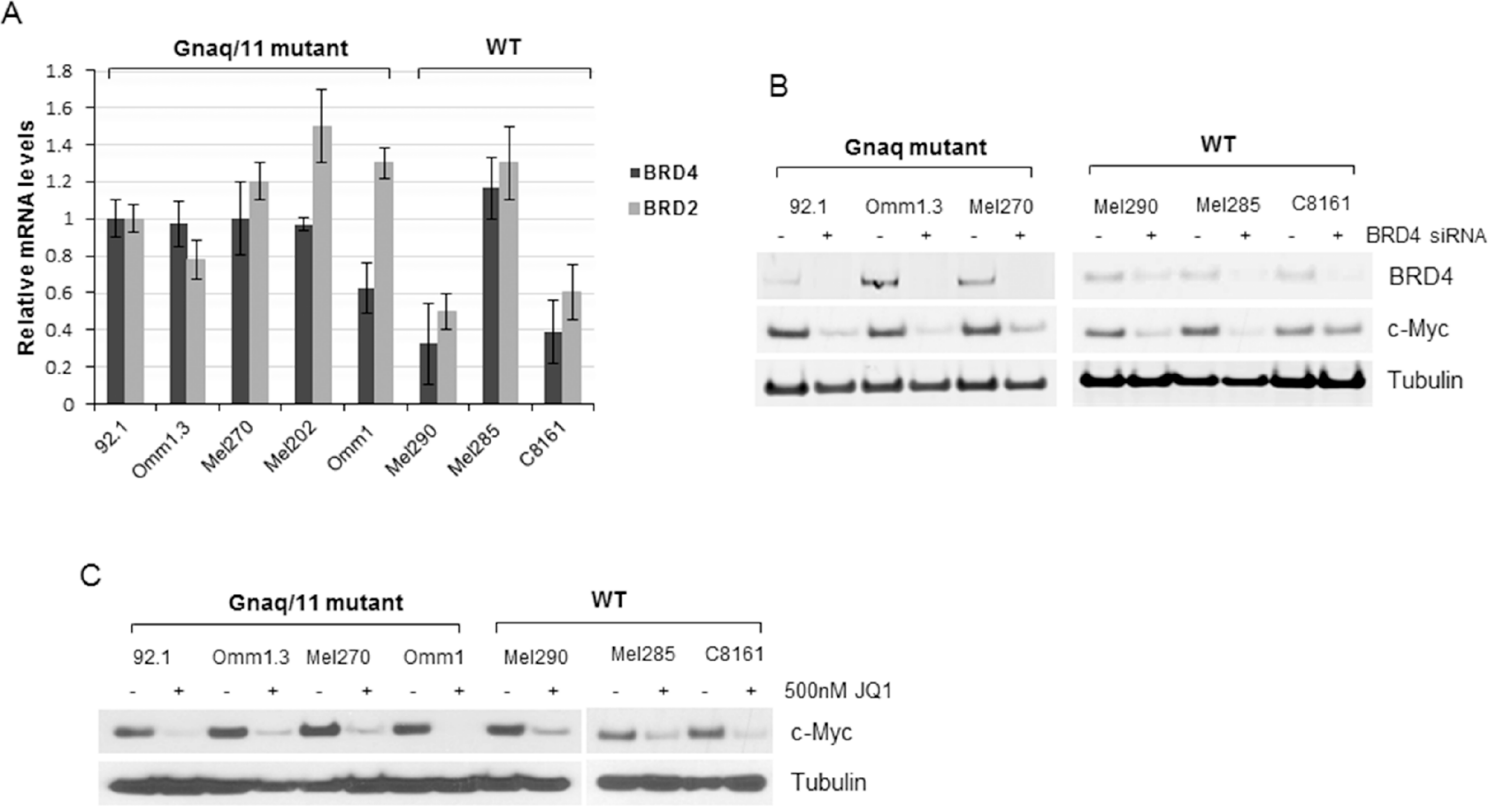
BRD4 and BRD2 are expressed in UM cells **A.** Total RNA was extracted from eight cell lines with the indicated mutational status, and qPCR was performed using gene-specific primers for BRD4 and BRD2. Values were normalized with GAPDH as housekeeping gene using the ΔΔCT method. Values are relative to mRNA levels of 92.1 cells set at 1. Each experiment was performed in triplicates. Bars, mean ± sd. **B.** BRD4 is active in UM cells. Silencing of BRD4 down-regulates c-Myc expression in Gnaq mutant- and WT cell lines, as detected by immunoblotting. **C.** Cells were treated with 500 nM JQ1 for 24 hours and cell lysates were analyzed for c-Myc expression and tubulin as loading control.

We first analyzed the status of *MYC* in UM cells by FISH analysis, and found that several cell lines had extra copies of *MYC*, and the cell line Mel290 had true *MYC* amplification. Furthermore, four cell lines carried Gnaq mutation (92.1, Omm1.3, Mel270, Mel202), one cell line carried Gna11 mutation (Omm1), while Mel285 and Mel290 had neither mutation, designed as wild-type (WT). We also included a cutaneous melanoma cell line, C8161, which has extra copies of *MYC*, and no Gnaq/11 mutations [25]. These cell lines were tested for expression of BRD4 and BRD2 by real-time PCR. Figure 1A shows that the level of expression of these two genes was similar in the Gnaq/11 mutant cell lines, while it varied among the non-mutant cells, with lower expression in Mel290 and C8161, and high RNA expression of both genes in Mel285.

In order to test whether BRD4 is active and regulates expression of c-Myc in these cells, BRD4 was knocked down by siRNA transfection, and c-Myc was analyzed by immunoblotting. Despite the differences in BRD4 mRNA expression, BRD4 silencing suppressed c-Myc protein in all the cell lines (Figure 1B), suggesting that BRD4 is functional in these cells. The exception was the cutaneous melanoma cell line C8161, in which c-Myc expression was slightly inhibited.

Next, we tested the effect of the BET inhibitor JQ1 on c-Myc expression. Treatment of the cells with 500 nM JQ1 for 24 hours showed suppression of c-Myc in all the cell lines (Figure 1C), demonstrating target inhibition of this oncogene.

We next screened the cell lines for sensitivity to JQ1 treatments in viability assays.

**Figure 2:**
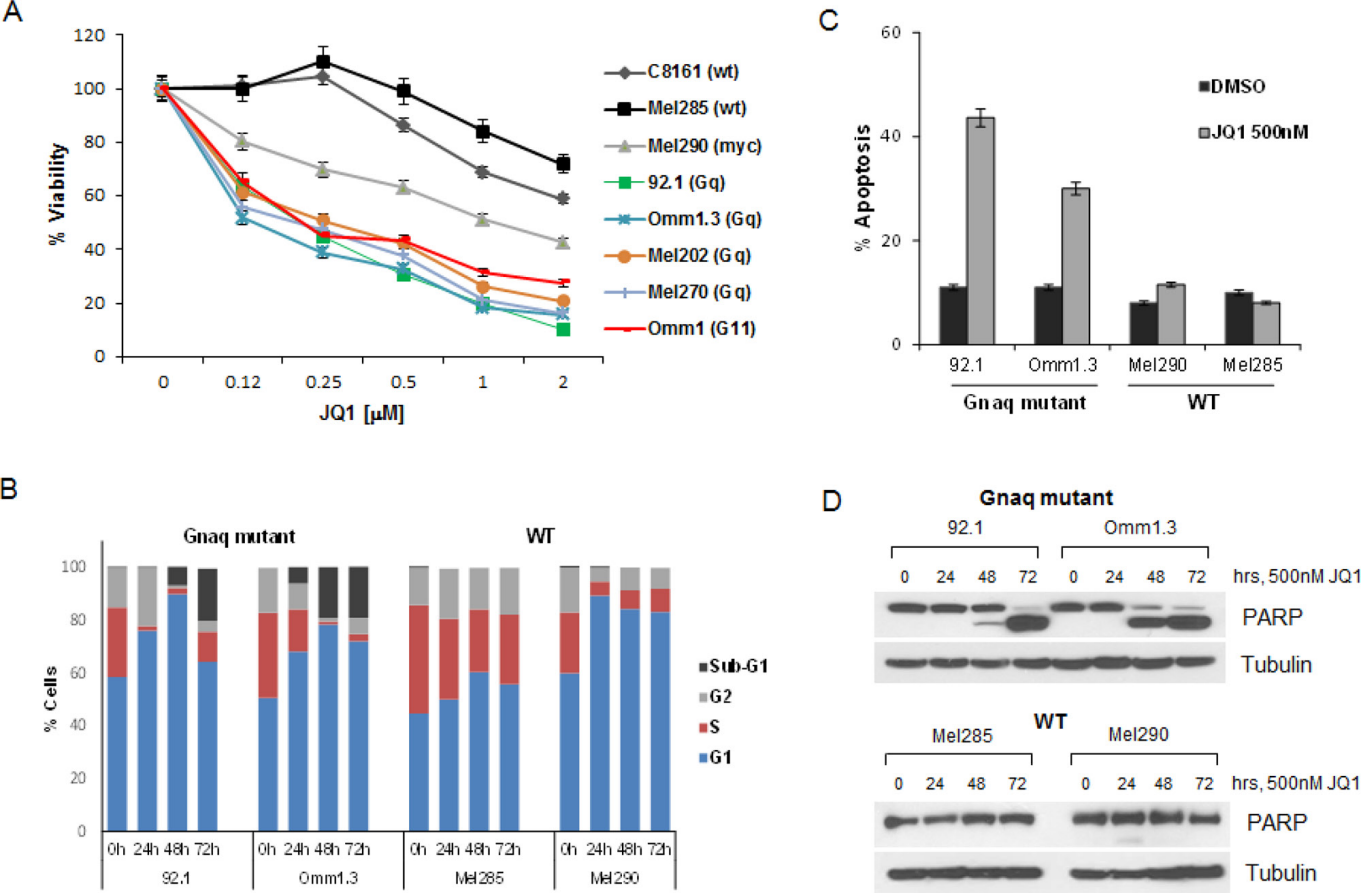
JQ1 induces cell cycle arrest and apoptosis in UM cells **A.** JQ1 reduces viability of a panel of UM cell lines with the indicated mutational status. The cell lines were exposed to 2-fold serial dilutions 2000–100 nM of JQ1 in triplicates for 4 days, and viability was normalized to DMSO-treated cells. Data points are mean ± sd. **B.** Gnaq-mutant and WT cell lines were treated with DMSO or 500 nM JQ1 over time up to 72 hours. The cells were stained with propidium iodide (PI) and analyzed for cell cycle distribution by flow cytometry. Sub-G1 populations were 19.8% and 19.2% for 92.1 and Omm1.3 cells, respectively. **C.** UM cells were treated with 500 nM JQ1 for 48 hours, then incubated with YO-PRO dye (green) and PI (red). Bars report the percent of cells with the sum of green and red fluorescence for each condition in triplicates ± sd. **D.** The same cell lines (Gnaq-mutant top panel; WT, bottom panel) were treated over time with JQ1 and lysed for Western blot analysis, showing induction of apoptosis by PARP cleavage.

Most cell lines showed reduced viability with increasing doses of JQ1 (Figure 2A). However, the cells with Gnaq/11 mutations were the most sensitive to the treatments with IC_50_ of 100–250 nM, suggesting a dependency on functional BET proteins. Surprisingly, the *MYC*-amplified cell line Mel290 was not as sensitive, and the cells without G-protein mutation or *MYC* amplification, Mel285 and C8161, were the least sensitive to JQ1 with IC_50_ values well above 2000 nM.

We further investigated the effect of JQ1 on the cell lines with different mutational status by analyzing cell cycle progression. All four cell lines underwent cell cycle arrest in G1 (Figure 2B), while a marked apoptotic sub-G1 peak appeared in the Gnaq mutant cells after 48 and 72 hours of treatment. No sub-G1 was detected in the WT cells at any time point. The induction of apoptosis was also measured with a membrane permeability assay after 48 hour treatment (Figure 2C). Only the Gnaq-mutant cell lines (92.1 and Omm1.3) underwent apoptosis with increased permeability of 43.6% and 33% of the cell population, respectively. Finally, apoptosis was detected in the Gnaq mutant cells by the induction of cleaved PARP, an apoptotic marker, after 48 and 72 hours of treatment (Figure 2D, upper panel), while no PARP cleavage was induced in the WT cells at any time point (Figure 2D, lower panel).

Thus, JQ1 regulates c-Myc expression in all UM cell lines, but triggers apoptosis only in a subset of cell lines, specifically cells carrying Gnaq/11 mutations.

Mutant Gnaq and Gna11 proteins have long been known to activate downstream signaling targets, including MEK, PI3-kinase/Akt and protein kinase C, and the combination of specific inhibitors of these pathways were reported to effectively block proliferation of UM cells [26–29]. We tested whether JQ1 had combinatory effects with specific inhibitors of MEK (selumetinib), PKC (sotrastaurin) or AKT (MK2206). The viability of cells treated with various concentrations of JQ1, alone or in combination with each drug (0 to 2000 nM) was evaluated in two Gnaq-mutant cell lines. The combinatorial treatments were analyzed with the Chou-Talalay method [30] and found not synergistic with either drug. Each combination had a “fractional activity” (Fa) < 0.5 and combination index (CI) values > 1. Viability graphs using equimolar concentrations of each drug are shown in Supplemental Figure S1. This data suggests that JQ1 has potent cytotoxic activity as single agent in Gnaq/11-mutant UM, and the addition of another selective inhibitor would not improve JQ1 efficacy *in vitro*.

### BET-inhibition regulates the expression of genes involved in the regulation of cell cycle, apoptosis and DNA repair response

In order to investigate the effect of BRD4 inhibition on gene expression, we performed transcriptional analysis of seven UM cell lines and one cutaneous melanoma cell line with different G protein mutational status. The cells were treated with DMSO or 500 nM JQ1 for 24 hours, and profiled by gene expression microarrays.

**Figure 3:**
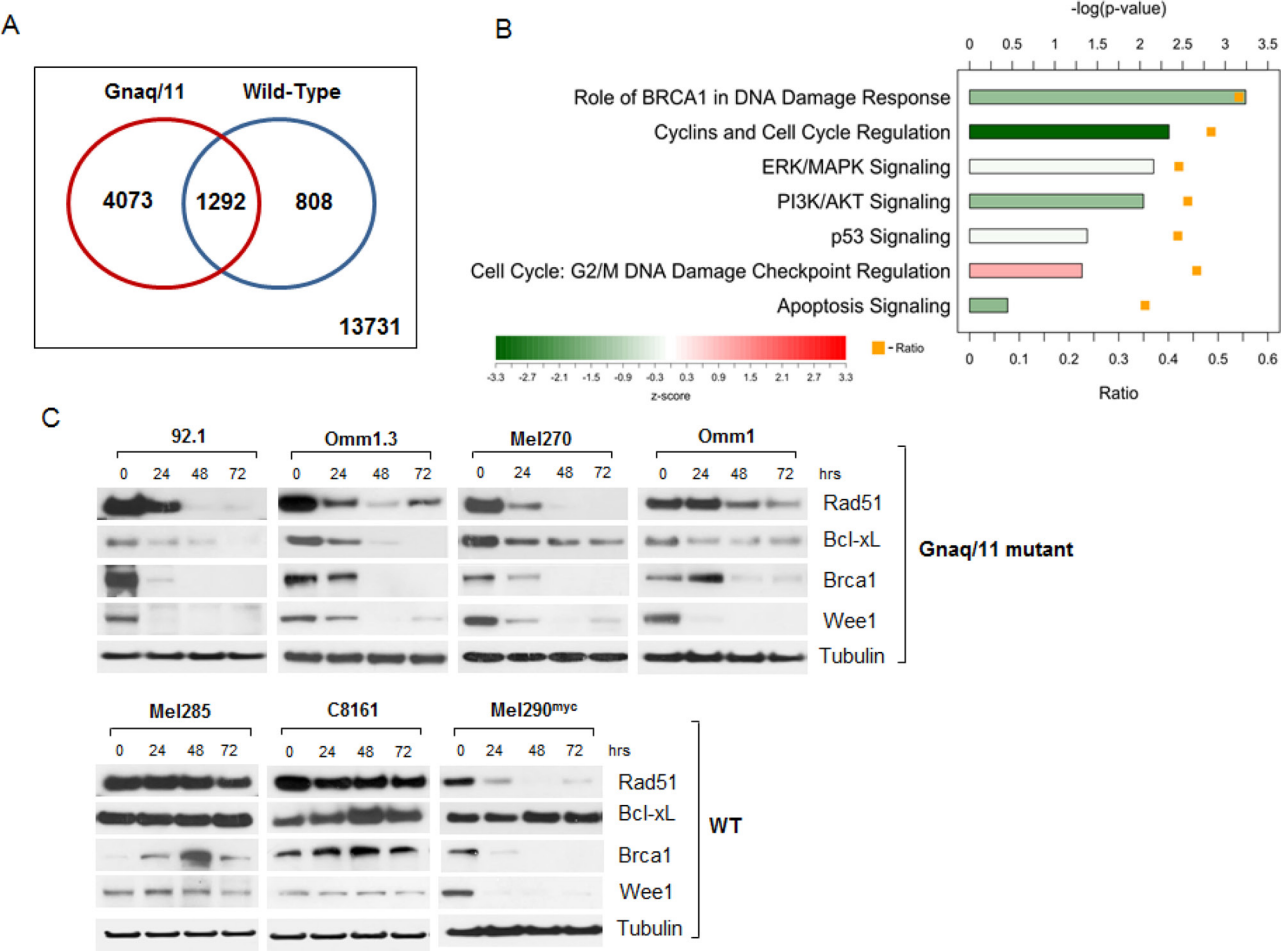
Microarray analysis of JQ1-treated cells reveals expression changes of numerous genes involved in signaling pathways, apoptosis and DNA repair **A.** Venn-diagram summarizing differentially expressed genes in JQ1-treated Gnaq/11-mutant cell lines (red circle), and WT cells (blue circle), with corresponding overlapping genes as indicated. Data is deposited at GEO accession no. GSE66048. **B.** Ingenuity Pathway Analysis for genes differentially expressed in Gnaq/11-mutant cells in response to treatment. The bars show the −log(*p*-value) from a Fisher's Exact Test for enrichment, and the color indicates the z-score for the pathway. The orange squares indicate the ratio of differentially expressed genes in the pathway that were differentially expressed in the mutant cell lines. **C.** Immunoblot analysis of UM cells with Gnaq/11 mutations (top panel) or without the mutations (WT, lower panel) treated with 500 nM JQ1 over time, using antibodies against the indicated proteins. Each blot is representative of at least 2 experiments showing same results.

The analysis of significant differentially expressed genes (*p* ≤ 0.05) identified 5365 transcripts that were regulated by JQ1 in Gnaq/11-mutant cells, while 2100 genes were regulated in the WT cells. This analysis included both up- and down-regulated genes. Data is deposited at GEO accession no. GSE66048. A comparison of genes regulated by JQ1 in the mutant versus WT cell lines, defined 4073 transcripts that were exclusively modulated in the Gnaq/11-mutant cells (Venn diagram, Figure 3A), while 1292 genes overlapped between the 2 groups. Thus, a much larger number of genes were regulated by JQ1 in the Gnaq/11 mutant cells, suggesting that BRD4 is particularly active in these cells lines.

To explore the biological relevance of differentially regulated genes in the Gnaq/11 mutant cells, we performed pathway analysis by Ingenuity (Figure 3B), which revealed several genes implicated in the regulation of cell cycle (i.e. *CCNE1, MYC, WEE1, E2F3, CDKN2D*), regulation of apoptosis (*BCL2L1, FOXO1*), and DNA damage response (*BRCA1, RAD51, CHEK1*). Several genes were similarly regulated by JQ1 in both Gnaq/11-mutant and WT cells, like *BCL2L11* (BIM), *CDKN1A* (p21/Cip1) and *BIRC5*. JQ1 treatment also affected genes involved in MAPK signaling (*DUSP4, ELK*) and PI3K/AKT pathway (*PIK3CB, AKT1, FOXO3*).

### Bcl-xL is a selective target of JQ1 in Gnaq/11 mutant cells

We have shown that MEK and ATK pathways are both activated in Gnaq/11 mutant cells [27, 31]. However, inhibition of one or both pathways induced cell cycle arrest and autophagy. Since JQ1 had potent anti-proliferative effects with induction of apoptosis, we selected genes that regulate the apoptotic pathway (BCL2L1, also called Bcl-xL), as well as cell cycle (c-Myc, Wee1), and the DNA damage response (Rad51 and Brca1) for further analysis. The regulation of these genes by JQ1 was confirmed at the protein level by immunoblotting following drug exposure for up to 72 hours. There was nearly complete inhibition of the proteins tested in cells with Gnaq/11 mutations (Figure 3C, top panel), while no inhibition was detected in the WT cell lines C8161 and Mel285 (Figure 3C, lower panel). The *MYC*-amplified cell line Mel290 was the exception, as most genes were also down-regulated, while Bcl-xL was not affected by the drug (Figure 3C).

**Figure 4:**
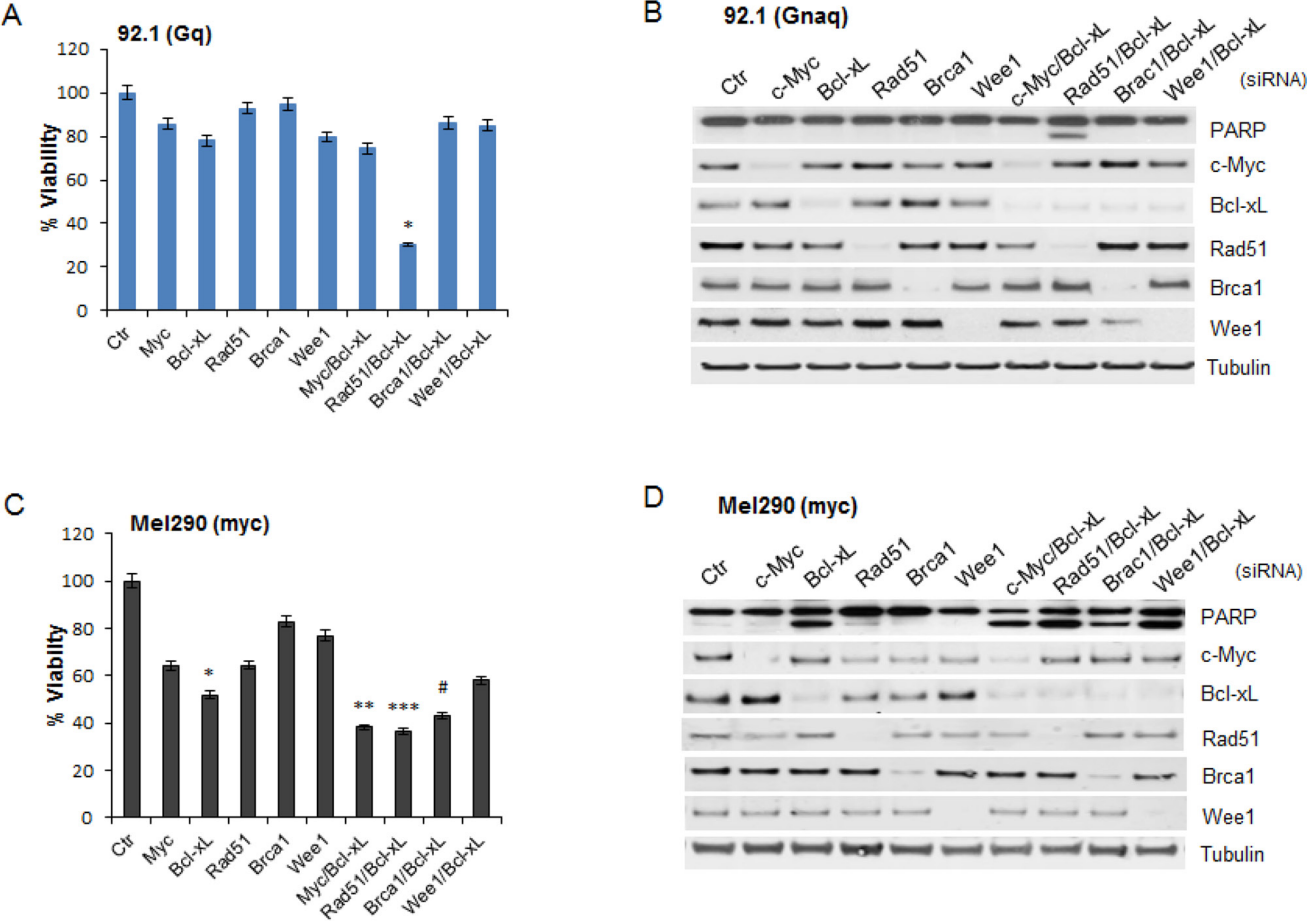
Effect of silencing of selected JQ1-regulated genes The indicated JQ1-regulated genes were silenced in the Gnaq-mutant cell line 92.1 (top) and in the myc-amplified cell line Mel290 (bottom). **A.** and **C.** siRNA transfected cells were plated in 96 well plates in triplicates and assayed for cell viability after 72 hours. Viability is calculated as percentage of cells transfected with a control siRNA (Ctr). Graphs are representative of three independent experiments. Bars, mean ± sd. *P < 0.001, **P < 0.01; ***P < 0.05, comparing the effect of gene-specific silencing versus control siRNA-transfected cells. The down-regulation of each gene was analyzed by immunoblotting for 92.1 **B.** and Mel290 cells **D.**

In order to determine whether any of these genes could mediate the apoptotic effects of JQ1, each gene was silenced by siRNA. Knockdown of single genes, including c-Myc, did not inhibit cell viability or induce apoptosis in the Gnaq-mutant cell line 92.1 (Figure 4A) and Omm1.3 (Supplemental Figure S2A and S2B). Surprisingly, the concomitant depletion of Bcl-xL and Rad51 significantly decreased viability of the cell lines 92.1 (Figure 4A) and Omm1.3 (Supplemental Figure S2B). In contrast, silencing of Bcl-xL together with c-Myc, Brca1 or Wee1 had no effect. The downregulation of each protein is shown in Figure 4B, and induction of PARP cleavage is detected only when Bcl-xL and Rad51 siRNA were combined, reproducing JQ1 effects.

In the *MYC*-amplified cell line Mel290, the depletion of c-Myc partially inhibited cell proliferation with no induction of apoptosis, similar to JQ1 treatment (Figure 4C). In contrast, Bcl-xL silencing alone or together with other gene-specific siRNA, induced a significant decrease in cell viability and PARP cleavage (Figure 4C and 4D).

None of these effects were detected in the WT cell line Mel285 with either siRNA transfection (Supplemental Figure S3A and S3B).

The induction of apoptosis by Bcl-xL and Rad51 depletion was also confirmed by using a different set of siRNA in the cell lines 92.1 and Mel290, obtaining a similar induction of apoptosis by PARP cleavage (Supplemental Figure S4).

Thus, Bcl-xL seems to play a role in cell survival of Gnaq/11-mutant and *MYC*-amplified cell lines, while c-Myc did not mediate the apoptotic effects of JQ1.

These findings would also suggest that although JQ1 inhibits expression of numerous genes, the concomitant down-regulation of Bcl-xL and Rad51 is the minimal requirement for inducing apoptosis in UM cells with Gnaq mutation.

**Figure 5:**
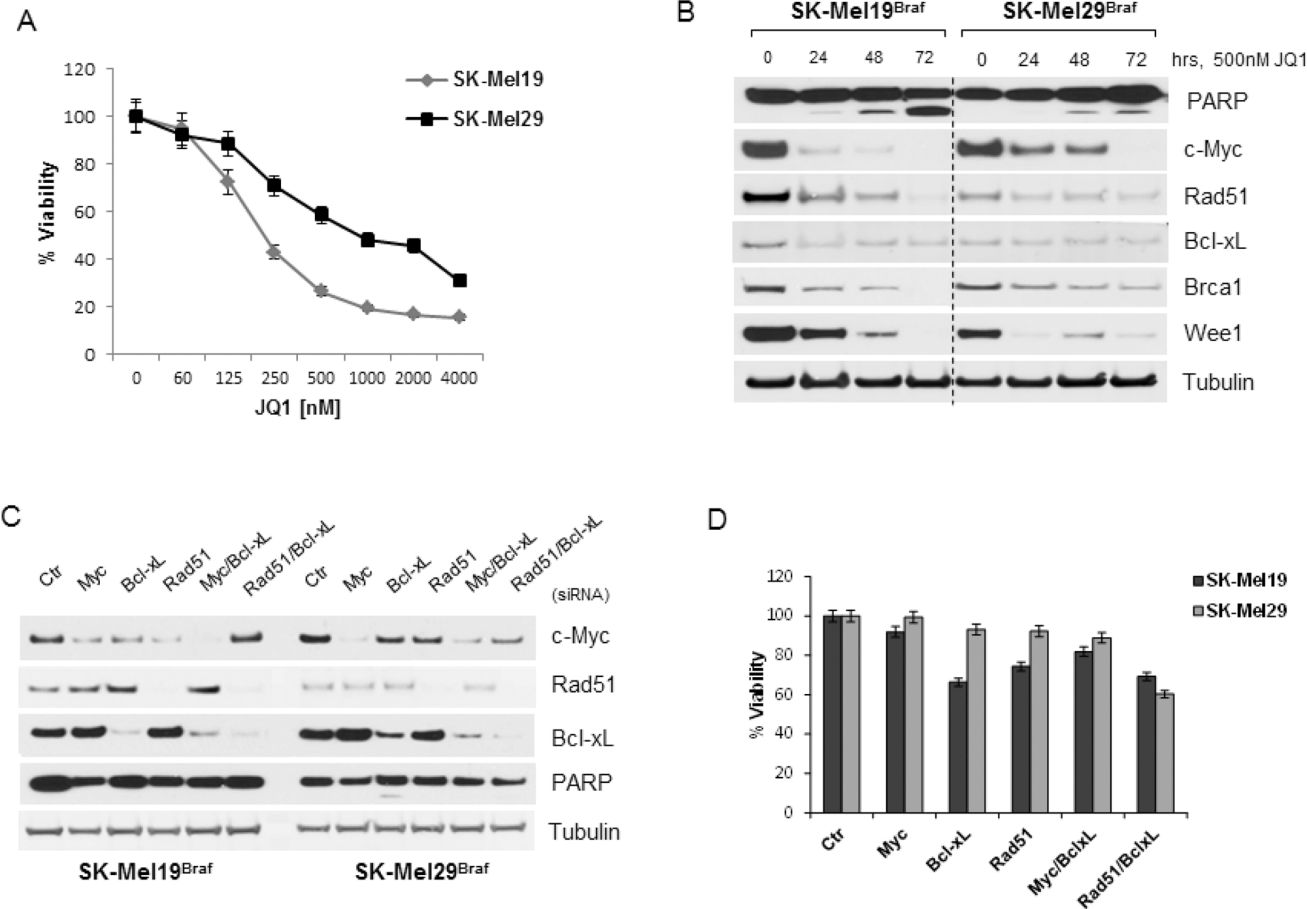
BRAF mutant melanoma cells are sensitive to JQ1 through other mechanisms **A.** Viability of BRAF-mutant cutaneous melanoma cells (SK-Mel19 and SK-Mel29) was assayed after 4 days of exposure to increasing doses of JQ1 treatments. **B.** Immunoblot analysis of BRAF-mutant cells treated with 500 nM JQ1 over time, using antibodies against c-Myc, Bcl-xL, Rad51, Brca1, Wee1 and PARP. **C.** The same genes were silenced and their expression was tested by immunoblotting. **D.** siRNA-transfected cells were tested for cell viability after 72 hours from transfection. Bars, mean ± sd

We sought to evaluate the importance of these genes in a comparable tumor, such as cutaneous melanoma with BRAF-mutation. The cell lines SK-Mel19 and SK-Mel29, carrying BRAF^V600E^ mutation, showed sensitivity to JQ1 in viability assays (Figure 5A) similar to UM Gnaq-mutant cells, with IC_50_ of 125–500 nM. JQ1 treatments also induced PARP cleavage and decreased the expression of c-Myc, Rad51, Brca1 and Wee1 in both cell lines, while Bcl-xL slightly decreased only in SK-Mel19 (Figure 5B). Silencing of the indicated genes (Figure 5C) did not induce inhibition of viability (Figure 5D), and the concomitant suppression of Bcl-xL and Rad51 had partial effects that were not statistically significant. These results suggest that genes like Rad51 and Bcl-xL specifically regulate UM cell survival, while in melanoma cells JQ1-induced apoptosis seems to be mediated by other mechanisms [32].

### JQ1 directly regulates Bcl-xL and Rad51 expression at the promoter region in Gnaq-mutant cells

Bcl-xL was identified in the microarray analysis among the genes involved in the regulation of apoptosis, and it was exclusively down-regulated by JQ1 in the Gnaq/11 mutant cells. It has been recently reported that Bcl-2 and Bcl-xL are highly expressed in UM, and their inhibition had antitumor activity [33]. Moreover, Rad51 was reported to have increased activity in cancer cells compared to normal cells [34]. Therefore, Bcl-xL and Rad51 may represent important mediators of cell survival in UM, and the expression of both genes is disrupted by BRD4 targeting.

**Figure 6:**
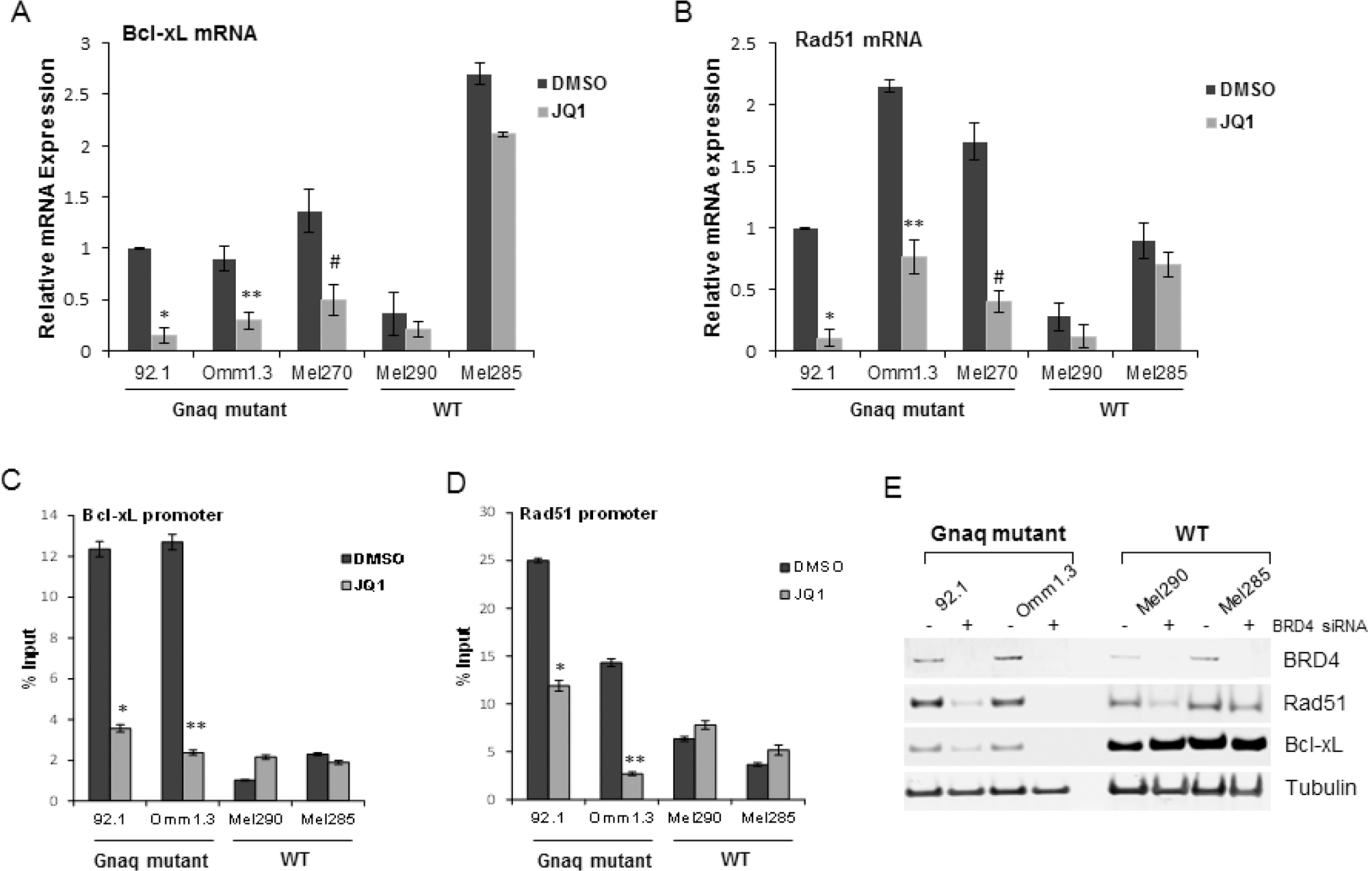
JQ1 directly suppresses Bcl-xL and Rad51 in Gnaq/11 mutant cells The effect of JQ1 on the mRNA of Bcl-xL and Rad51 was confirmed by qPCR in UM cell lines. Total RNA was extracted from cells with different mutational status after 24 h of treatment with 500 nM JQ1, and qPCR was performed using gene-specific primers for Bcl-xL **A.** and Rad51 **B.** Values were normalized with GAPDH as housekeeping gene using the ΔΔCT method. Values are relative to mRNA levels of 92.1 untreated cells set at 1. Each experiment was performed two or three times in triplicates. Bars, ± sd. *, **P < 0.01; #*P* < 0.05, comparing treatment versus DMSO. BRD4 ChIP assay for the Bcl-xL promoter **C.** and Rad51 promoter **D.** presented as percent of input, before and after treatment. Bars are representative of two independent experiments ± sd. *P < 0.001; **P < 0.01; #, *P* < 0.05. **E.** Downregulation of BRD4 regulates Bcl-xL and Rad51 expression in the Gnaq-mutant cells. The indicated cell lines were transfected with a non-specific siRNA (−) or BRD4 siRNA (+), and analyzed by immunoblotting with the indicated antibodies.

To confirm suppression of the mRNA levels of these genes by JQ1, quantitative RT-PCR was performed in Gnaq/11-mutant and WT cells before and after JQ1 treatment. Bcl-xL was significantly suppressed in the Gnaq/11-mutant cells (Figure 6A, and Supplemental Figure S5A), while its basal expression was much higher in the WT cell lines Mel285 and C6181, and minimally affected by JQ1. The *MYC*-amplified cell line Mel290 had low levels of Bcl-xL mRNA and the decrease induced by JQ1 was not significant (Figure 6A). Rad51 was also markedly down-regulated by JQ1 in the Gnaq/11-mutant cells (Figure 6B and Supplemental Figure S5B), while no significant inhibition was detected in all WT cells.

Next, we asked whether Bcl-xL and Rad51 promoters were directly regulated by BRD4. Using chromatin immunoprecipitation assay (ChIP), we found that BRD4 was enriched at the Bcl-xL promoter in the Gnaq-mutant cells (Figure 6C), and the treatment with JQ1 diminished this binding. In contrast, there was minimal binding to the Bcl-xL promoter in the WT cell lines, and JQ1 did not change it. A similar displacement of BRD4 binding by JQ1 was found at the Rad51 promoter in the mutant cells (Figure 6D), but not in the WT cells.

Finally, we sought to demonstrate that BRD4 depletion could specifically down-regulate these two proteins. BRD4 silencing caused a decrease of both Bcl-xL and Rad51 expression in the Gnaq-mutant cells (Figure 6E). Instead, BRD4 depletion caused a slight decrease in Rad51, but not Bcl-xL, in the Mel290 WT cells. No changes were detected in Mel285, suggesting that Bcl-xL and Rad51 are regulated by other mechanisms in these cells.

Taken together these data indicate that BRD4 is required for Bcl-xL and Rad51 expression, and JQ1 inhibits BRD4 recruitment to their promoters in the Gnaq mutant cells.

**Figure 7:**
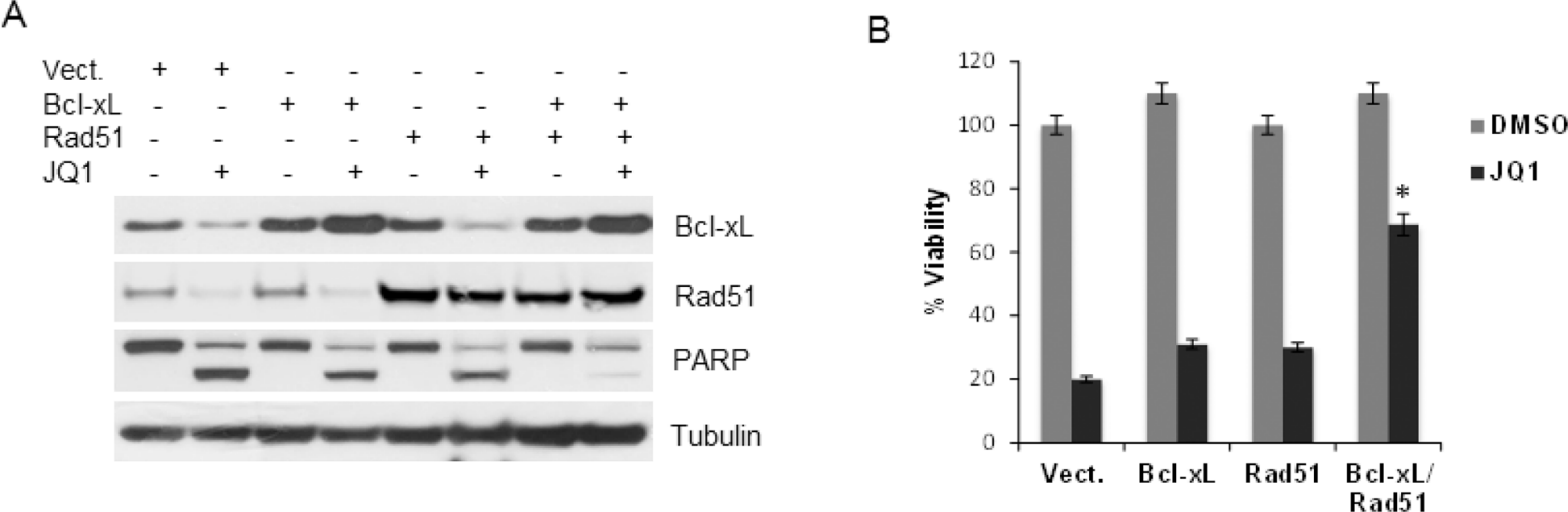
Overexpression of Bcl-xL and Rad51 partially protects cells from JQ1-induced cytotoxic effects **A.** 92.1 cells were transfected with an empty vector (pcDNA3), Bcl-xL and Rad51 alone or together, before JQ1 treatment. Cell lysates were subject to immunoblotting using Bcl-xL, Rad51, PARP and tubulin antibodies. **B.** Viability assay of transfected cells with or without treatment with JQ1. Columns, mean of three independent experiments. ± sd. *P = 0.003 comparing the effect of JQ1 in cells overexpressing Bcl-xL and Rad51 versus vector-transfected cells.

Because JQ1 caused down-regulation of Bcl-xL and Rad51, we assessed whether the overexpression of these two proteins could protect UM cells from JQ1. Bcl-xL was ectopically expressed alone or together with Rad51 in the mutant cells, and protein levels are shown in Figure 7A. Neither Bcl-xL nor Rad51 alone could rescue cells from JQ1 treatment in cell viability assays (Figure 7B). However, when both constructs were expressed there was a significant increase in cell survival in the presence of JQ1 (Figure 7B), which also corresponded to a decrease of cleaved PARP (Figure 7A).

Although numerous genes are affected by JQ1, our results suggest that these two proteins play an important role in mediating BET-mediated regulation of UM survival.

### JQ1 inhibits tumor growth *in vivo* in a Gnaq-mutant xenograft model

**Figure 8:**
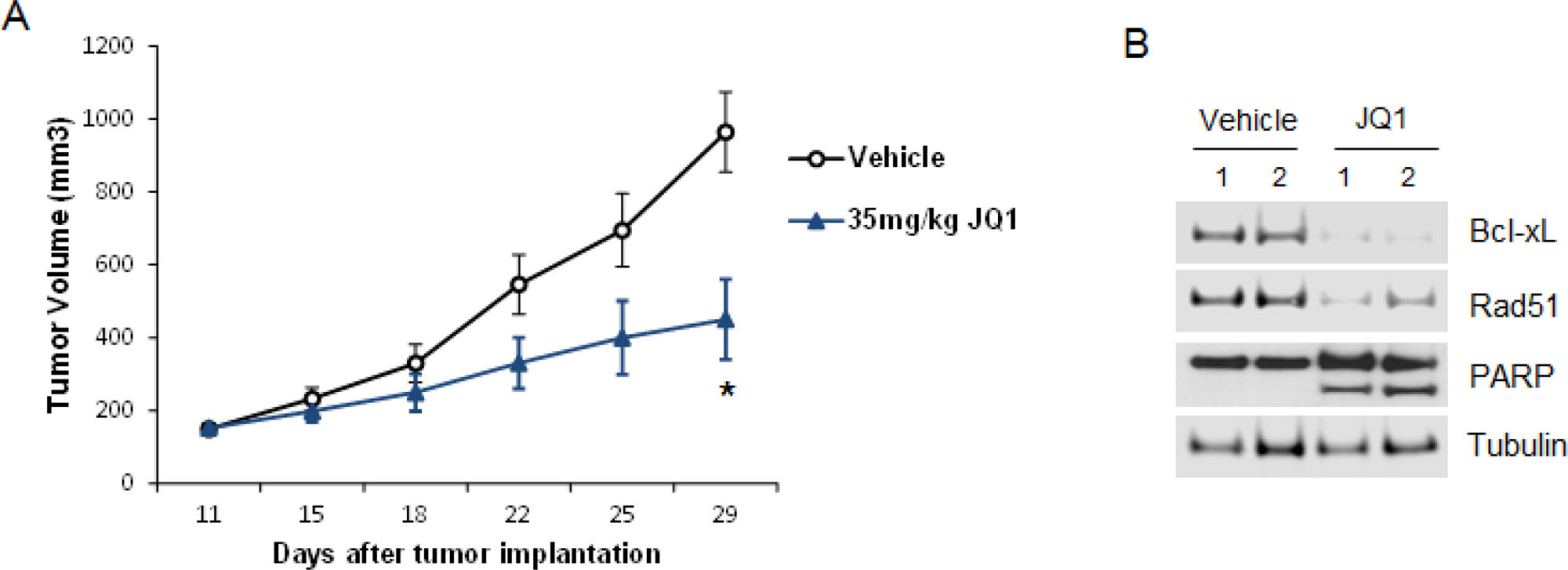
JQ1 inhibits UM tumor growth *in vivo* **A.** JQ1 inhibited tumor growth in a xenograft model with the Gnaq-mutant 92.1 cell line. Six- to eight-week SCID female mice were subcutaneously injected with 92.1 cells. Drug treatments began after tumors reached 100 mm^3^. Mice bearing tumors were treated daily with JQ1 (35 mg/kg) orally for 5 days each week for a total of 3 weeks. Tumors were measured with calipers every 2 to 3 days and tumor volumes were compared between groups of mice at various points in time. Each value represents the mean measurement of 5 animals, ± SEM, *P < 0.05. **B.** Xenograft tumors were collected at the end of treatments from two vehicle- and two JQ1-treated mice, and analyzed by Western blotting with antibodies for Bcl-xL, Rad51 and PARP.

The therapeutic potential of JQ1 was also analyzed *in vivo*. We established a xenograft model of UM in mice using a Gnaq-mutant cell line. JQ1 was administered orally at 35 mg/kg, five times a week for three weeks. The dose was well tolerated as no body weight loss due to the treatment was detected up to a month from the first dose. Tumor growth was inhibited in the treated mice compared to vehicle (Figure 8A), and this inhibition was significant after 21 days of treatment (*p* < 0.05). In addition, immunoblotting of proteins from tumors collected at the end of the treatments showed down-regulation of Bcl-xL and Rad51 (Figure 8B). We could also detect induction of apoptosis by PARP cleavage, confirming the effects of JQ1 observed in UM cell lines *in vitro*.

## DISCUSSION

Previous studies have demonstrated that extra copies of *MYC* are present in UM [22, 35], which would predict sensitivity to BRD4 inhibition.

Here we report that the BET inhibitor JQ1 induces cell cycle arrest in a panel of genetically diverse UM, and it has potent cytotoxic effects only in cells with Gnaq/11 mutations, irrespective of *MYC* status. Transcriptional microarray analysis of these cells treated with JQ1 revealed that a greater number of genes are susceptible to regulation by JQ1, compared to cells without Gnaq/11 mutations. A subset of these genes, selected from canonical pathway enrichment analysis and common to all mutant cell lines, was further analyzed by gene silencing. These studies determined that the suppression of each gene, including c-Myc, was insufficient to mimic the apoptotic effects of JQ1. Rather, the concomitant depletion of Bcl-xL and Rad51 induced apoptosis similar to JQ1, suggesting that the simultaneous regulation of multiple effectors is necessary for the induction of apoptosis in these cells. While the down-regulation of both genes represented the minimal requirement to mimic JQ1 effects, we cannot rule out the possibility that other genes may be responsible for mediating the apoptotic effects of BET inhibition, as JQ1 treatment led to down- and up-regulation of numerous genes with potential roles in tumorigenesis and cell survival. Given the high incidence of *MYC* amplification in UM and its correlation with larger tumor size [24], c-Myc targeting may still represent a possible strategy to manage this disease.

Interestingly, Bcl-xL was downregulated by JQ1 only in the Gnaq/11-mutant cells, but not in the less sensitive WT cells. Although Bcl-xL was slightly down-regulated by JQ1 in one BRAF-mutant cell line, it did not seem to play a role in mediating the apoptotic effects of JQ1 in these cells. On the other hand, knockdown of Bcl-xL was sufficient to induce cell death in the *MYC*-amplified cell line, confirming that Bcl-xL expression is critical for UM survival. Regulation of Bcl-xL by JQ1 was also reported in glioblastoma [12], and its over-expression partially rescued cells from JQ1-induced apoptosis.

Recent studies have identified other important targets of JQ1, such as FosL1 in lung cancer cell lines [11], or IL7R in lymphoblastic leukemia [10]. The cytotoxic effects of bromodomain inhibition reported in cutaneous melanoma cells were independent of the mutational status of BRAF or NRAS [14]. This is in contrast with the effect of JQ1 in UM cell lines, where the induction of apoptosis was dependent on Gnaq/11 mutations, suggesting that these cells rely on BRD4 activity for the regulation of gene transcription. This is of particular interest, as nearly 85% of UM carry Gnaq/11 mutations, and BRD4 inhibition could translate in targeted therapies for the majority of UM.

The regulation of DNA repair genes such as Rad51 by JQ1 also contributed to the survival of cells with Gnaq mutation. BRD4 was recruited to the promoter of Rad51 and Bcl-xL, and was displaced by JQ1 in the mutant cells, confirming regulation of expression of this gene by BET-proteins. The differential effects of JQ1 in the genetically diverse UM could have several plausible explanations. Although BRD4 is a ubiquitous regulator, it also has gene-specific effects due to the presence of super-enhancer in tumor cells. The enhancers function through cooperative and synergistic interactions between multiple transcription factors and coactivators [36, 37], which confer increased transcription of target genes and higher sensitivity to specific inhibitors. Further investigation will be required to determine whether these mechanisms are responsible for the differential binding of BRD4 to chromatin in different tumors and cell lines.

BET inhibitors have been reported to have cytostatic effects in several tumor types, requiring combinations with other drugs to induce synergistic effects [32, 38]. In contrast, drug combinations may not be necessary for the treatment of Gnaq/11-mutant UM, as JQ1 caused marked cell death as single agent, and the combinations with other small molecules targeting mutant Gnaq signaling did not have synergistic effects.

A number of BET bromodomain inhibitors are now under development, and some of them (i.e. OTX015 and GSK525762) are in phase I clinical studies. These drugs have shown gene expression signature with a large overlap with JQ1 expression profiling [12, 38, 39], and they may represent effective therapies against UM in the clinical setting.

In summary, our results determine that BET inhibition represents an effective therapy against UM through the inhibition of cell cycle and induction of apoptosis *in vitro* and *in vivo*. This data strongly supports the rationale for the targeting of BRD4 in patients with UM harboring Gnaq/11 mutations.

## MATERIALS AND METHODS

### Cell lines and reagents

The cell lines Omm1.3, Mel270, Mel202 were kindly provided by Dr Bruce Ksander, Harvard Medical School, Boston, MA. 92.1 cells have been provided by Dr William Harbour, Washington University, St. Louis, MO). Omm1 and Mel285 were provided by Dr Boris Bastian, University of California, San Francisco, CA. Mel290 and C8161 were from Dr Robert Folberg (University of Illinois, Chicago, IL). All the cell lines have been sequenced for the presence of activating mutations in codons 209 (exon 5) and 183 (exon 4) of Gnaq and Gna11. C8161 cells were recently characterized as cutaneous melanoma [25]. SK-Mel19 and SK-Mel29 were a gift from Dr Taha Merghoub (Memorial Sloan-Kettering Cancer Center, New York, NY). Cells were cultured in RPMI medium supplemented with 10% fetal bovine serum, 100 units/ml penicillin and 100 μg/ml streptomycin, and maintained at 37°C in 5% CO_2_. FISH was performed using red-labeled probe from PAC clone RP1–80K22 spanning *MYC* in 8q24, together with green-labeled centromeric probe (pJM128) as reference. FISH signals were analyzed in a minimum of 10 metaphase spreads and 100 interphase nuclei. The cell lines 92.1, Omm1.3, Mel270 and Mel202 had extra copies of normal chromosomes 8, and *MYC* was highly represented. This is consistent with a previous analysis of the cell line 92.1 [40]. The cell line C8161 showed two copies of an isochromosome for 8q, with probable matching loss of 8p, giving a total of 7 copies of *MYC*. Mel290 cells showed amplification of *MYC*. Omm1 cells had normal *MYC* copy number. JQ1 was kindly provided by Dr James Bradner (Harvard Medical School, Boston, MA). Selumetinib, sotrastaurin and MK2206 were purchased from Selleck Chemicals.

### Cell viability assays

Cells were plated in 96-well plates, and treated with the indicated concentrations of drugs. Viability was assessed after four days of treatment using the Cell Counting Kit 8 (CCK8) from Dojindo Molecular Technologies according to the manufacturer's instructions. Cell viability is expressed as a percentage of untreated cells. Flow cytometry of cells was performed after staining with 5 μg/ml propidium iodide containing 50 μg/ml RNase A. Samples were analyzed on a FACScan (Becton Dickinson), and data were analyzed for DNA content using Flowjo software. Membrane permeability and apoptosis were measured using the YO-PRO-1 Kit (Life Technologies). Fluorescent cells were analyzed on a Cellometer K2 (Nexcelom Bioscience) with De Novo software.

### Microarray and statistical analysis

The cells were treated in triplicate with medium containing 0.2% DMSO or 500 nM JQ1 for 24 hours. Total RNA was extracted using the RNeasyMini kit (Qiagen). Samples were profiled using the genechip Affymetrix Human Gene Expression Array (HG-U133 2.0, Affymetrix) using established protocols. All statistical analyses were carried out in R using packages provided within Bioconductor [41]. Data were normalized using GC-RMA. Analysis of gene expression was performed using the limma package [42]. Differential gene expression was modelled using a blocked linear model design to account for between cell line effects, and correlation term to account for technical replication within cell lines [42]. Three contrasts were made to extract statistics for differential gene expression due to the effect of treatment on the wild type cell lines, the effect of treatment on the mutant cell lines and the difference in the effect of treatment between mutant and wild-type cell lines. False discovery rate (FDR) was estimated using the method described by Benjamini and Hochberg [43]. Statistical significance of gene expression was determined at FDR ≤ 0.05. Gene lists of interest were exported from R and analyzed for the enrichment of biological pathways using Qiagen's Ingenuity Pathway Analysis. Data is deposited at GEO accession no. GSE66048.

### Immunoblotting

Cells were lysed in RIPA buffer (Cell Signaling) supplemented with protease inhibitor cocktail tablets (Roche Diagnostics). Total protein concentration of the lysates was measured by BCA assay (Bio-Rad), and equal amounts of protein were loaded on 4–12% PAGE gels (Life Biotechnologies). PVDF membranes were blocked with 5% nonfat dried milk in PBS buffer containing 0.1% Tween-20 and probed with antibody for c-Myc, PARP, Bcl-xL, Brca1, BRD4, tubulin (Cell Signaling), Rad51 and Wee1 (Santa Cruz Biotechnology). Signals from secondary antibodies were detected using ECL (Pierce) and autoradiography films (Fisher Scientific) or using the Odyssey scanner (LiCOR Biosciences).

### siRNA and plasmid transfections

Small interfering RNA against c-Myc (sc-29226), Rad51 (sc-36361), Brca1 (sc-29219), Wee1 (sc-36835) and control siRNA (sc-37007) were purchased from Santa Cruz Biotechnology. Bcl-xL siRNA was from Cell Signaling (#6362), and siRNA-2 SMARTpool from Dharmacon (L-004937-00). siRNA-2 for Rad51 (L-003530-00) and BRD4 (L-004937-00) were also from Dharmacon. They were transfected using Lipofectamine RNAiMAX reagent (Life Technologies). Rad51 construct [44] and Bcl-xL (OriGene) were transfected using Fugene 6 (Promega), following manufacturer's instructions.

#### Quantitative real-time PCR

Total RNA was reverse-transcribed using the SuperScript III First Strand System (Life Technologies). The resultant cDNA was used in qPCR reactions using 7500 Real Time PCR System (Applied Biosystems) with pre-designed TaqMan Gene expression assays for BRD4, BRD2, Bcl-xL, Rad51 and glyceraldehyde-3-phosphate dehydrogenase (GAPDH) genes (Life Technologies). Triplicates CT values were averaged and normalized to GAPDH. The relative expression of each gene was calculated by the ΔΔCT method. Statistical significance was determined by 2-sample Student *t* tests.

#### Chromatin Immunoprecipitation Assay (ChIP)

Cells were cross-linked with 1% formaldehyde and then quenched by 0.125 M glycine. Cells were then harvested and washed, and nuclear extraction was performed using the SimpleChip Enzymatic ChiP Kit (Cell Signaling) following the manufacturer's instructions. Equivalent amounts of chromatin from each sample were then immunoprecipitated with the BRD4 antibody overnight at 4°C. Antibody-protein complexes were then collected using Protein G agarose beads (Cell Signaling) pre-blocked with salmon sperm. Eluted DNA was reverse cross-linked, treated with proteinase K, and purified. Immunoprecipitated DNA and input controls were then analyzed on an Applied Biosystems 7500 real-time PCR machine, using Taq SYBR Green (Life Technologies) and primer sets for Bcl-xL promoter, (forward) 5′-GGGAGTGGTCTTTCCGAA-3′, and (reverse) 5′-CTCCATCGACCAGATCGA-3′. Primers for the Rad51 promoter were from Qiagen.

#### Animal studies

Severe combined immunodeficient mice (SCID) mice were purchased from Taconic, and used when they were 8-weeks old. 92.1 cells were inoculated subcutaneously into the right flanks of the mice. When tumors reached a volume of approximately 100 mm^3^ diameter, animals were administered (5/group) vehicle or JQ1 35 mg/kg orally. The treatment duration was 3 weeks and the tumor size was measured twice a week. After the fifth treatment, two animals from each cohort were sacrificed and the tumors were collected for Western blot analysis. Experiments were carried out under an Institutional Animal Care and Use Committee–approved protocol, and Institutional guidelines for the proper and humane use of animals were followed. Statistical significance was determined by 2-sample Student *t* tests.

## SUPPLEMENTARY FIGURES


