# Supplementary material for: BRD4-targeted therapy induces Myc-independent cytotoxicity in Gnaq/11-mutatant uveal melanoma cells
Source: Oncotarget. 2015 Sep 5;6(32):33397–409. doi: 10.18632/oncotarget.5179 (PMC4741774; doi:10.18632/oncotarget.5179)
Supplement: Supplementary file 1 [file oncotarget-06-33397-s001.pdf]

SUPPLEMENTARY FIGURES

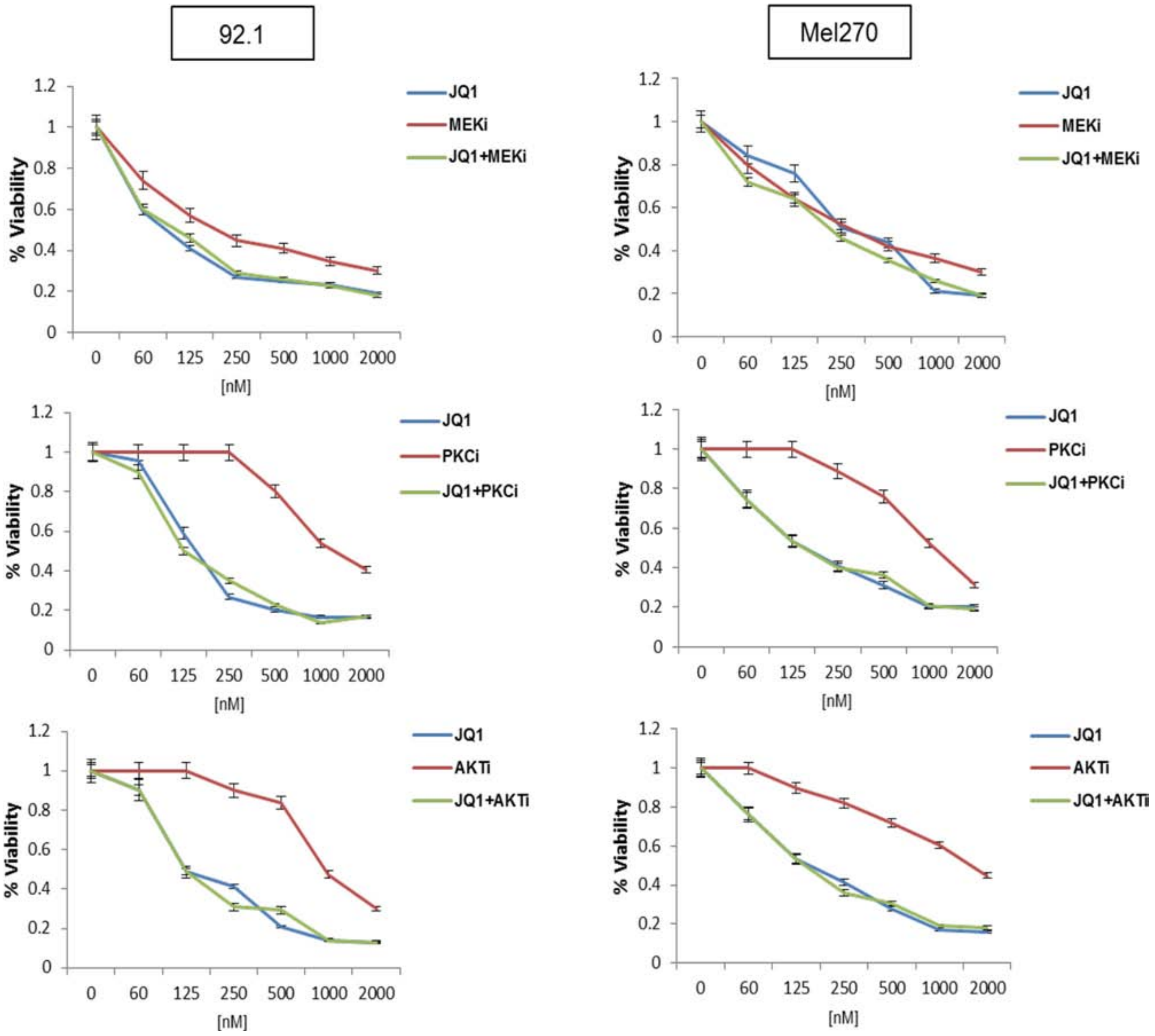

Supplementary Figure S1: Gnaq-mutant cell lines 92.1 (left) and Mel270 (right) were treated with increasing concentrations of JQ1 (0 to 2000 nM) alone or in combination with selumetinib (MEKi), sotrastaurin (PKCi) or MK2206 (AKTi) (0 to 2000) for 4 days. Cell viability is expressed relative to untreated cells set at 1. Each experiment was performed in triplicates ± sd.

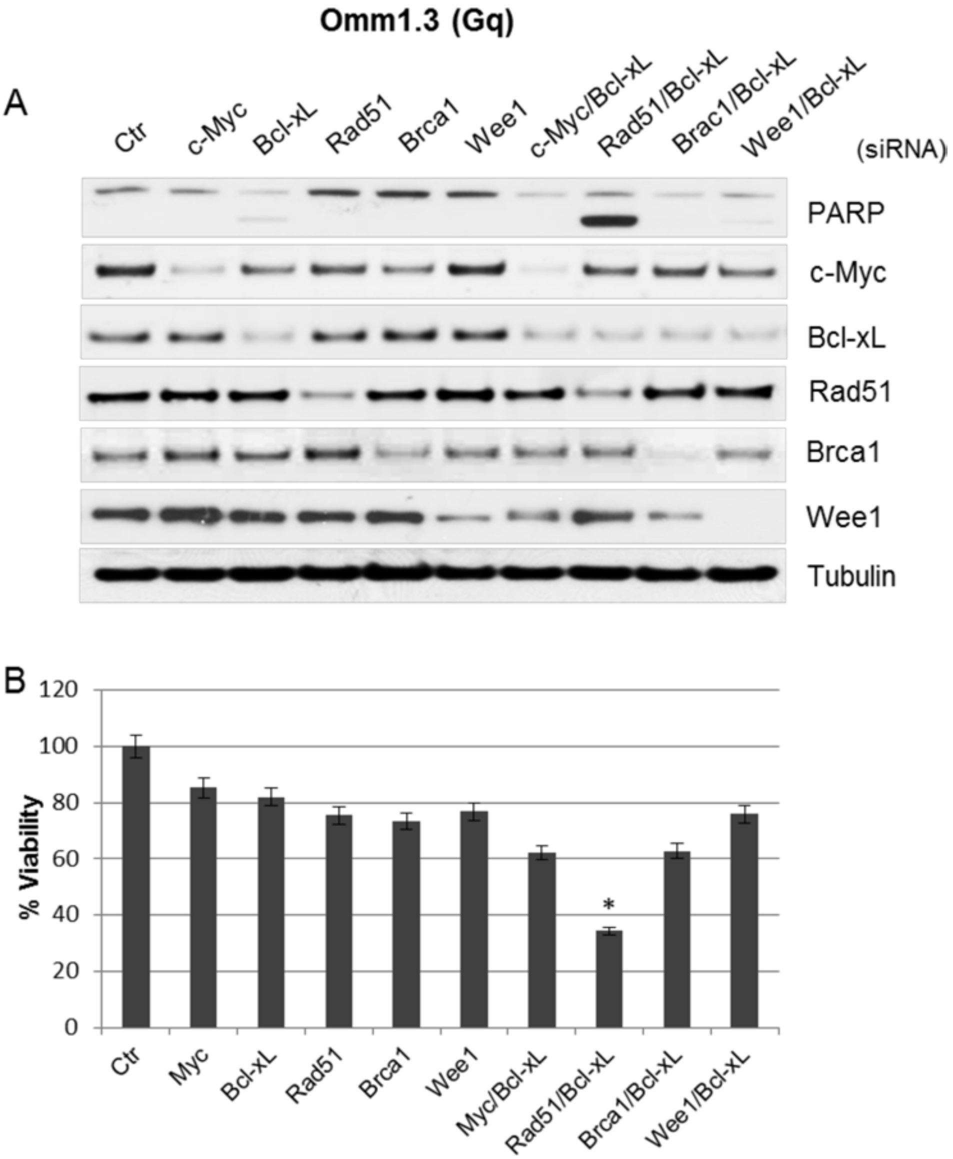

Supplementary Figure S2: A. SiRNA-mediated knockdown of the indicated genes in the Gnaq-mutant cell line Omm1.3. PARP cleavage is induced only after concomitant silencing of Bcl-xL and Rad51. **B.** Cell viability was measured after three days from siRNA transfection. The silencing of Bcl-xL and Rad51 significantly decreases cell viability. Experiments were repeated three times in triplicates ± sd. *$p < 0.05$

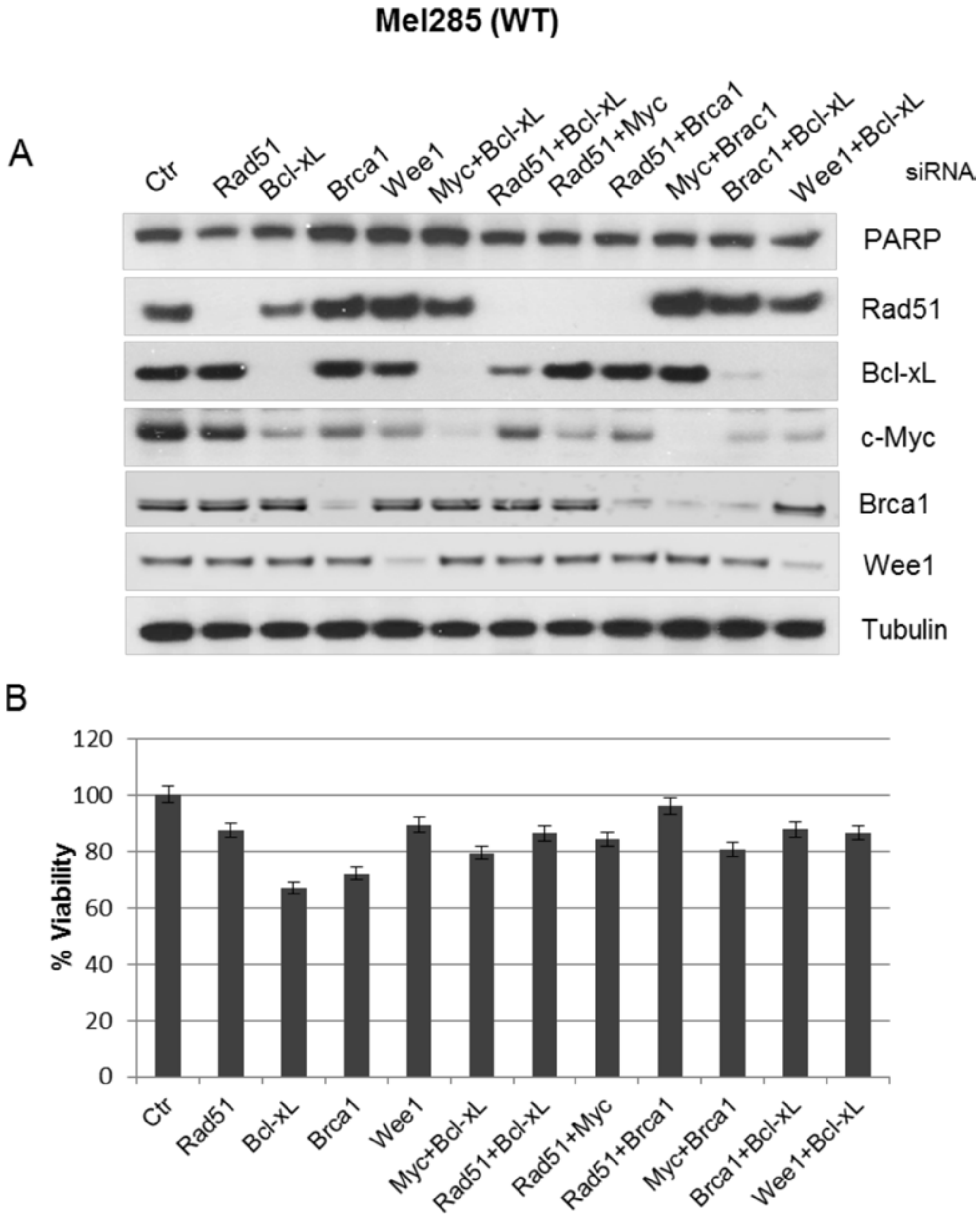

Supplementary Figure S3: siRNA-mediated knockdown of the indicated genes in the wild-type cell line Mel285 did not induce PARP cleavage. A. or a decrease of cell viability **B.** after either siRNA transfection.

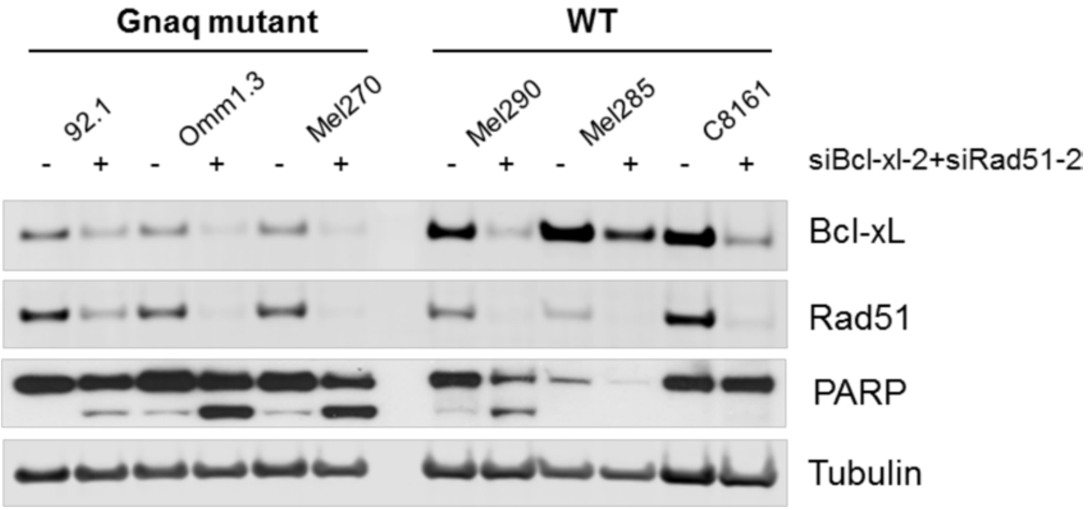

Supplementary Figure S4: Mutant Gnaq and WT cells were transfected with a different set of siRNA-2 (+) for Bcl-xL and Rad51 to confirm specific downregulation of the two proteins in the indicated cell lines. A non-specific siRNA (−) was used as a control. There was induction of PARP cleavage in the mutant cells and in the myc-amplified cell line Mel290, but not in the WT cells.

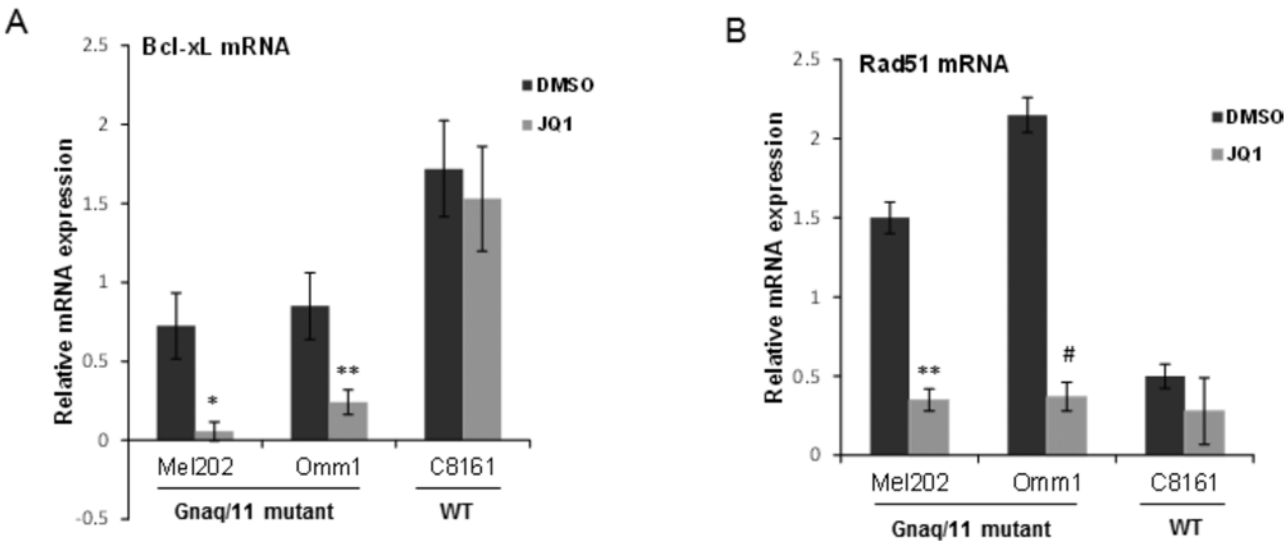

Supplementary Figure S5: Total RNA was extracted from UM cells and a CM cell line with different mutational status after treatment with 500 nM JQ1 for 24 h, and qPCR was performed using gene-specific primers for Bcl-xL A. and Rad51 **B.** Values were normalized to GAPDH as housekeeping gene using the ΔΔCT method, and are relative to the mRNA levels of untreated 92.1 cells, set at 1. Each Experiment was performed in triplicates. Bars, ± sd. *p** < 0.001; ** < 0.01; # = 0.05
